# Anti-Fungal Analysis of *Bacillus subtilis* DL76 on Conidiation, Appressorium Formation, Growth, Multiple Stress Response, and Pathogenicity in *Magnaporthe oryzae*

**DOI:** 10.3390/ijms23105314

**Published:** 2022-05-10

**Authors:** Veronica Tshogofatso Kgosi, Bao Tingting, Zhao Ying, Hongxia Liu

**Affiliations:** 1 Department of Plant Pathology, College of Plant Protection, Nanjing Agricultural University, Nanjing 210095, China; tshegofatso.vero@yahoo.com (V.T.K.); 2019102028@njau.edu.cn (B.T.); nongda2020@163.com (Z.Y.); 2 Key Laboratory of Integrated Management of Crop Diseases and Pests, Nanjing Agricultural University, Ministry of Education, Nanjing 210095, China

**Keywords:** *Magnaporthe oryzae*, *Bacillus subtilis* DL76, conidiation, multiple stress tolerance, pathogenicity

## Abstract

In recent years, biological control has gained more attention as a promising method to combat plant disease. Such severe diseases cited include rice blasts caused by *Magnaporthe oryzae*. However, more effective microbial strains with strong adaptability still need to be identified. Therefore, we sought to assess the conidia germination, and formation of appressorium of DL76 in *Magnaporthe oryzae*. Besides, we also aimed at understanding the growth, multiple stress response and pathogenicity in *Magnaporthe oryzae*. We isolated *Bacillus subtilis* DL76 from a rice farm, which observed a strong antimicrobial effect on *M. oryzae*. The sterilized culture filtrate of DL76 inhibited the growth of *M. oryzae*, which motivated us to deduce the influence of DL76 on the pathogenicity of *M. oryzae*. We screened the effect of *Bacillus subtilis* DL76 on *M. oryzae* guy11. It demonstrated that sterilized culture filtrate (1 × 10^7^ CFU/mL) of DL76 can delay and even suppress the germination of conidia and (1 × 10^7^ and 1 × 10^6^ CFU/mL) prevent the formation of appressorium in vitro and in vivo. DL76 became hypersensitive to osmotic, oxidative, and cell wall degrading agents. In addition, the relative transcript levels of stress-responsive genes oxidative and osmotic were down-regulated by DL76 except for *sod1*, *cat1*, and *cat2*. In vivo assessment of the antifungal activity of *Bacillus subtilis* using conidia suspension of DL76 reduced the incidence and severity of rice blast. Conclusively, our results show that DL76 is essential for controlling rice blast by inhibiting conidiation, growth, multiple stress tolerance, and pathogenicity in *M. oryzae.*

## 1. Introduction

*Magnaporthe oryzae* is one of the most destructive pathogens of rice worldwide, causing a significant yield loss [1]. Outbreaks of rice blast disease are a serious and recurrent problem in China and other rice-growing regions. Therefore, several farmers use antifungal pesticides and plant disease-resistant cultivars to reduce the loss caused by rice blasts [2]. However, physiological races can quickly evolve and adapt to the resistant rice varieties because of the complexity of rice blasts. It is also challenging to control rice blast by planting resistant rice varieties because of their genetic background. Moreover, the overuse of chemical fungicide pollutes the environment and enhances the drug resistance in rice blasts amidst other advantages it offers [3]. 

Biological control has emerged as an environmentally friendly and promising strategy to control plant pathogens due to the increasing focus on sustainable development in agriculture [4]. Recent literature projects an increasing interest in exploiting rice blasts for biological control using beneficial plant micro-organisms because of their low toxicity and a lack of pathogen resistance [5,6,7]. Biological control is more beneficial than chemical fungicides because it is simple, highly efficient, and has less environmental impact. As a result, it has been widely used for micro-organism control, including *Bacillus spp*., Actinomycetes, *Pseudomonas spp*. and other anti-fungal bacteria that produce a series of antibiotics and enzymes [8]. Bacillus is considered a biological control candidate in agriculture because of its high antifungal properties and resistance to extreme conditions. *Bacillus spp*. has attracted significant attention because it exists universally in nature and can produce a series of antifungal substances as a non-pathogenic bacterium. Bacillus can increase plant resistance to pathogenic fungi by colonizing plants or spraying a plant with its powder, exerting a biological control effect [9,10,11]. Besides, *Bacillus* also promotes growth and increases plants’ yield, equating its biological control as immeasurable. 

Researchers have assessed the interactions between biocontrol agents and pathogens in rice and other commercially important crops [12,13]. Particularly, Yan et al. [14] explored the interactions between fungi and bacteria in rice and observed a competitive superiority between *Burkholderia gladioli* and *Aspergillus flavus* strains. Kumar et al. [15] analyzed interactions between *Bacillus subtilis* MBI 600 and *Rhizoctonia solani* and concluded that the MBI 600 strain suppressed the plant pathogen. Previous studies have demonstrated the antifungal activity of *Pseudomonas fluorescens* 2137 in suppressing *Fusarium culmorum* on barley roots [16]. The anti-fungal properties of Bacillus species, including *B. subtilis*, have been investigated for their biological control against many plant and animal diseases [17]. Bacillus strains act directly or indirectly to control plant pathogens by colonizing plants, forming bio-films, enhancing the provision of nutrients and phytohormones, or inducing systemic resistance.

For instance, *Bacillus amyloliquefaciens* has potential applications in the biological control of *Colletotrichum dematium* which causes Mulberry anthracnose. Therefore, it is crucial and urgent to identify more effective and safer methods to suppress rice blasts. As a result, we isolated *Bacillus subtilis*, DL76, from the rice rhizosphere in a field. The outcome indicated that it suppressed the conidia germination and appressorium formation with a biocontrol efficiency of 75% against *Magnaporthe oryzae*. In addition, our results suggested that DL76 is more sensitive to oxidants, osmotic and cell wall perturbing agents. Our study presents *B. subtilis* DL76 as a potential biocontrol agent against *M. oryzae*, the rice blast pathogen. 

## 2. Experiment Results

### 2.1. The Antifungal Activities of the Culture Filtrates DL76 at Different Concentration

**Figure 1:**
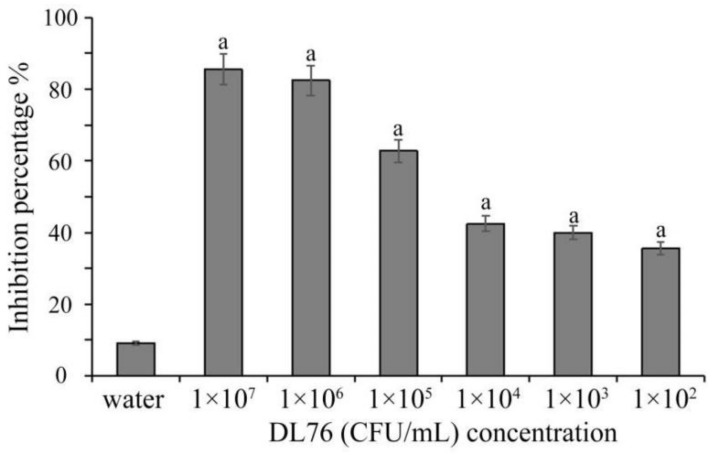
Effect of different concentrations on the suppression of *M. oryzae*. The formula of relative inhibition rate was described as Relative inhibition (%) = [(K_1_ − K_2_)/K_1_] × 100. Lower-case letters represent significant difference (*p* < 0.05).

According to the dual culture experiment, the endophytic bacteria DL76 showed salient antifungal activity against *M. oryzae*. Endophytic micro-organisms have been researched for decades as a potential source of biologically active metabolites in biological control [18]. Endophytic bacteria have been reported to have excellent anti-fungal activities. For instance, Gao et al. [19] identified an endophyte with significant antifungal activity against various fungi. The culture filtrate and fermentation broth of DL76 observed a significant suppression of *M. oryzae* growth at all concentrations (1 × 10^2^, 1 × 10^3^, 1 × 10^4^, 1 × 10^5^, 1 × 10^6^, 1 × 10^7^ colony-forming units (CFU/mL) (Figure 1). The inhibition rate ranges from 65–85 CFU/mL. That is, the suppression activity increased as the concentration increased. The outcome could propose that strain DL76 strongly inhibits *M. oryzae.*

### 2.2. Stability of Antifungal Metabolites

**Figure 2:**
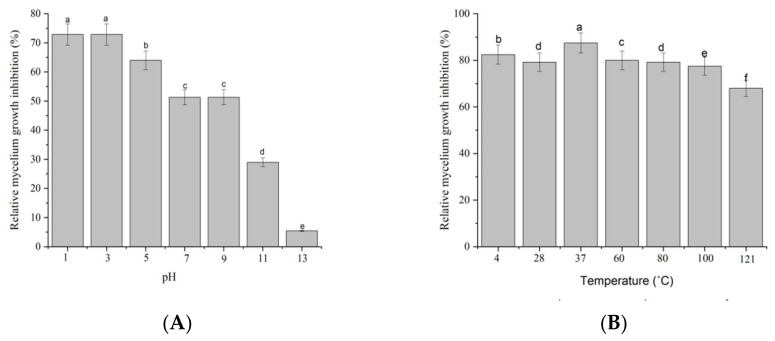
Antifungal activity of the pH-treated (**A**) and heat-treated (**B**) DL76 against *M. oryzae.* Error Bars represent the SEs of three replicates. Different letters indicate significant differences (*p* < 0.05) based on one-way analysis of variance followed by the least significant difference test.

The outcome from the acid-base stability assay observed a decline in the anti-fungal activity of culture filtrate DL76 after exposure to alkaline conditions at a pH of 11 or higher. The remaining inhibition for DL76 was significantly (*p* < 0.05) reduced to 29%, and 5.4% of the original level after alkali treatment at a pH of 11 and 13, respectively. However, the antifungal activity observed no significant difference at pHs between 1 and 9. The finding illustrated that the antifungal activity of the culture filtrate of DL76 against *M. oryzae* Guy11 was vital under strong acid, neutral and weak alkalinity conditions. Yet, the suppression activity diminished with the increase of alkaline (Figure 2A). Additionally, the antifungal properties exhibited by strain DL76 culture filtrate showed high thermal stability. The suppression rate reached 70% when the filtrate temperature ranged between 4 °C to 100 °C, reducing the suppression rate as the temperature increased (Figure 2B). The antifungal activity remained strong between 4 °C to 100 °C for 30 min but significantly decreased at 120 °C. Other studies, such as [20] observed similar results where *Bacillus spp*. produced antifungal metabolites for thermal stability and strong base instability. Therefore, high stability is necessary for developing anti-fungal metabolites as biocontrol pesticides. The fundamental stability of the anti-fungal metabolites produced by strain DL76 shows its potential in various environmental conditions.

### 2.3. The Correlation between Cell Growth and Anti-Fungal Activity

**Figure 3:**
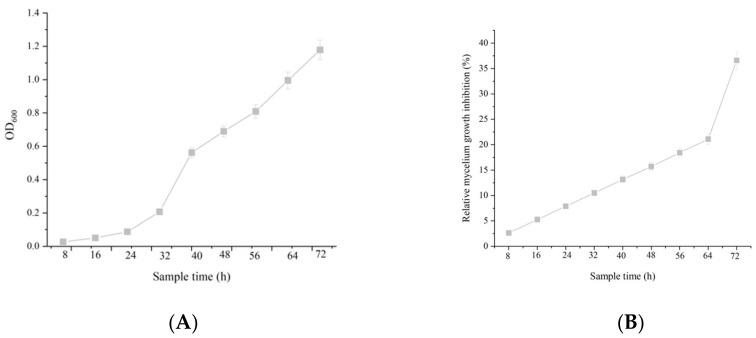
Correlation between cell growth and anti-fungal activity Correlation between strain DL76 growth and its inhibition percentage. (**A**) is means of the growth curve measured by OD600 and (**B**) indicates that antifungal activities of the sterilized culture filtrate of DL76 against *M. oryzae*. Relative inhibition (%) = [(K_1_ − K_2_)/K_1_] × 100.

Based on the experiment, the concentration of DL76 observed a positive correlation with the growth time. Growth curve analysis indicated that strains DL76 entered the logarithmic phase after 8 h of culture and reached the stationary state at 40 h. Further, the growth trend showed an S curve with increased cultivation time and concentration of DL76. The DL76 observed the fastest reproduction at 8 h. Nevertheless, there was no indication of the decline phase during the 72 h of incubation (Figure 3A). Similarly, there was a significant correlation between the culture filtrate bioactivity against *M. oryzae* and culture time. The anti-fungal activity increased rapidly over the entire logarithmic phase with the multiplication of bacteria (Figure 3B). Even more, we detected the strongest anti-fungal activity after forty-hours post-inoculation. Based on these findings, we could deduce that the optimal culture time to obtain strong anti-fungal metabolites occurs after forty hours. Various bacteria can produce anti-fungal metabolites, although they need a long fermentation time. Yet, strain DL76 can multiply and produce anti-fungal metabolites in a short time. However, DL76 can multiply and produce anti-fungal metabolites faster, thus it is convenient for production and application.

### 2.4. Effect of DL76 on Conidium Attachment

**Figure 4:**
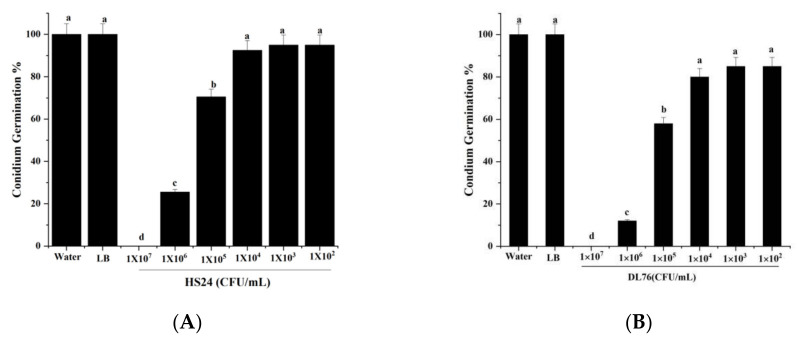
Effect of *Bacillus subtilis* DL76 at various concentrations on conidium germination at 8 h post-incubation. *Magnaporthe oryzae* conidia were treated with sterile water, Luria–Bertani (LB) broth, *Bacillus cerus* HS24, *Bacillus subtilis* DL76 at a concentration of 1 × 10^7^ CFU/mL. (**A**) represents conidium germination HS24, and (**B**) represents conidium germination DL76. Values are means and standard deviations of three replicates. Means separation letters indicate statistical significance (*p* < 0.05) based on one-way analysis of variance test. Scale bar = 30 µm.

After treating conidium germination by HS24 at 10^5^ CFU/mL, we observed an insignificant difference from the controls at 24 hpi. Germination increased from 17.9% at 8 hpi to 56.0% at 24 hpi when treated with HS24 at 10^6^ CFU/mL. A few conidia (2.1%) germinated when treated with HS24 at 10^7^ CFU/mL at 24 hpi (Figure 4A). The germination rate was 70% when DL76 10^5^ CFU/mL was treated at 24 hpi. Additionally, the 10^6^ CFU/mL germination rate increased from 12.5% at 8 hpi to 41.7% at 24 hpi (Figure 4B). Therefore, based on these results, we can conclude that the conidium germination gap between HS24 and DL76, indicates significantly different components of secondary metabolites between them. Hence, DL76 at the concentration of 1 × 10^7^ CFU/mL can significantly inhibit, delay, or even suppress the germination of conidia in *M. oryzae.*

### 2.5. The DL76 Suppresses the Formation of Appressorium

**Figure 5:**
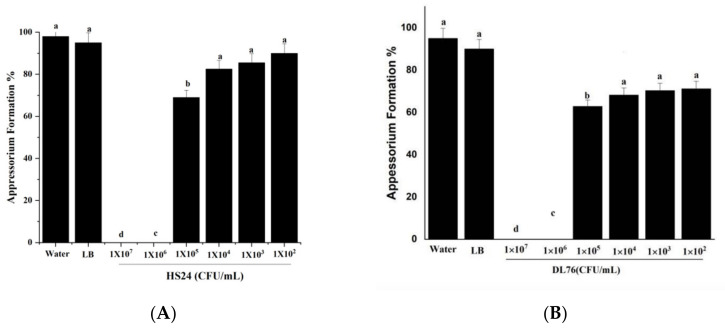
Effect of *Bacillus subtilis* DL76 at various concentrations on Appressorium formation at 8 h post-incubation. *Magnaporthe oryzae* conidia were treated with sterile water, Luria–Bertani (LB) broth, *Bacillus cerus* HS24, *Bacillus subtilis* DL76 at a concentration of 1 × 10^7^ CFU/mL (a negative control of bacterium). (**A**) represents Appressorium germination HS24, (**B**) represents Appressorium germination DL76. The values are means and standard deviations of three replicates. The means separation letters indicate statistical significance (*p* < 0.05) based on one-way analysis of variance test. Scale bar = 30 µm.

An appressorium is the typical penetration structure that helps the rice blast invade the host cell [21,22]. Figure 5B observes the outcome when DL76 is assayed for appressorium formation on hydrophobic surfaces at 1 × 10^7^ CFU/mL, eight hpi. We observed similar results using HS24 at the same concentrations. However, after incubating for 8 h on hydrophobic surfaces, we observed that over 95% of wild-type and water germ tubes formed appressoria, as presented in Figure 5A,B. An increase in concentration resulted in a suppression of appressorium formation. In contrast, germlings developing appressoria were formed with a lesser concentration. Similarly, DL76 at 1 × 10^5^ CFU/mL allowed 79% of the germlings to develop appressoria, and in a lower concentration of 1× 10^3^ and 1 × 10^2^ CFU/mL, over 85% appressorium formation was observed in the germ tubes, as illustrated in Figure 5B. The findings denote that at the concentration of 1 × 10^7^ and 1 × 10^6^ CFU/mL, most appressorium formation is delayed or inhibited, which is a critical infection structure for the pathogenicity ability of *M. oryzae*.

### 2.6. Differentiation of Appressoria

**Figure 6:**
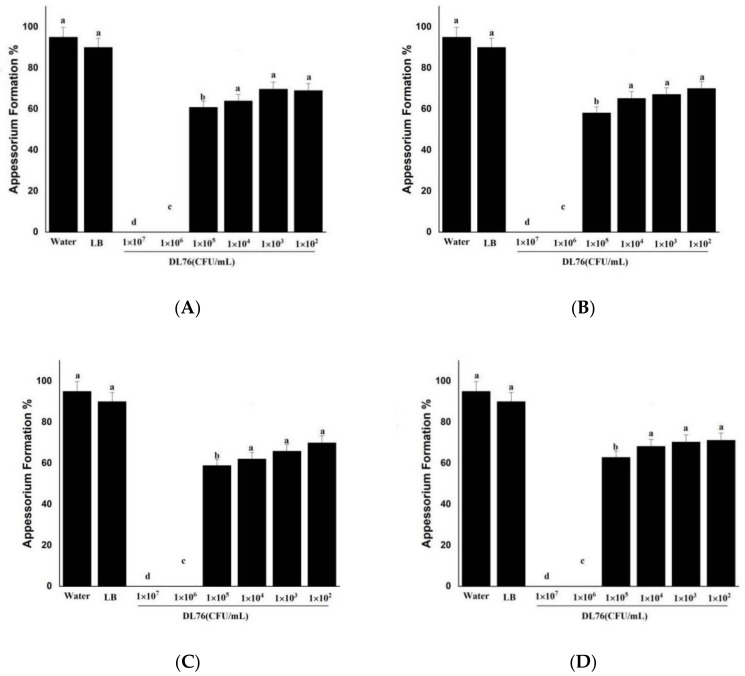
Effect of *Bacillus subtilis* DL76 on Appressorium formation in *Magnaporthe oryzae* at different time points. Conidia were treated with Luria–Bertani (LB) broth, sterile water, (control), or DL76 at the concentrations of 1 × 10^2^, 1 × 10^3^, 1 × 10^4^, 1 × 10^5^, 1 × 10^6^ or 1 × 10^7^ CFU/mL. (**A**) is the conidium germination evaluated with LB broth, sterile water, or DL76 at 24-h post-inoculation (hpi). (**B**) refers to conidium germination evaluated with LB broth, sterile water, or DL76 at 36-h post-inoculation (hpi). (**C**) refers to conidium germination evaluated with LB broth, sterile water, or DL76 at 48-h post-inoculation (hpi). (**D**) is the conidium germination evaluated with LB broth, sterile water, or DL76 at 60-h post-inoculation (hpi). The values are means and standard deviations of three replicates. The means separation letters indicate statistical significance (*p* < 0.05) based on one-way analysis of variance followed by the least significant difference test. Scale bar = 20 µm.

We further examined germlings for differentiation of the appressoria after 24, 36, 48, and 60 h of incubation on hydrophobic surfaces when most of the water and wild-type germlings had produced appressoria (Figure 6A–D). However, there was no observation of appressoria at 1 × 10^7^ and 1 × 10^6^ CFU/mL because the suppression of appressorium weakened with the decrease in the concentration of DL76. The appressorium formation developed significantly with more incubation time at a lower 1 × 10^2^ and 1 × 10^3^ CFU/mL concentration. Additionally, there were no significant changes in turgor pressure between water and DL76-treated *M. oryzae*. We observed a delayed appressorium formation by DL76 at 1 × 10^7^ and 1 × 10^6^ CFU/mL because almost 0% of the germlings had not produced appressoria after 24, 36, 48, and 60 h.

### 2.7. We Treated M. oryzae Conidia with Environmental Stress Inhibitors

**Figure 7:**
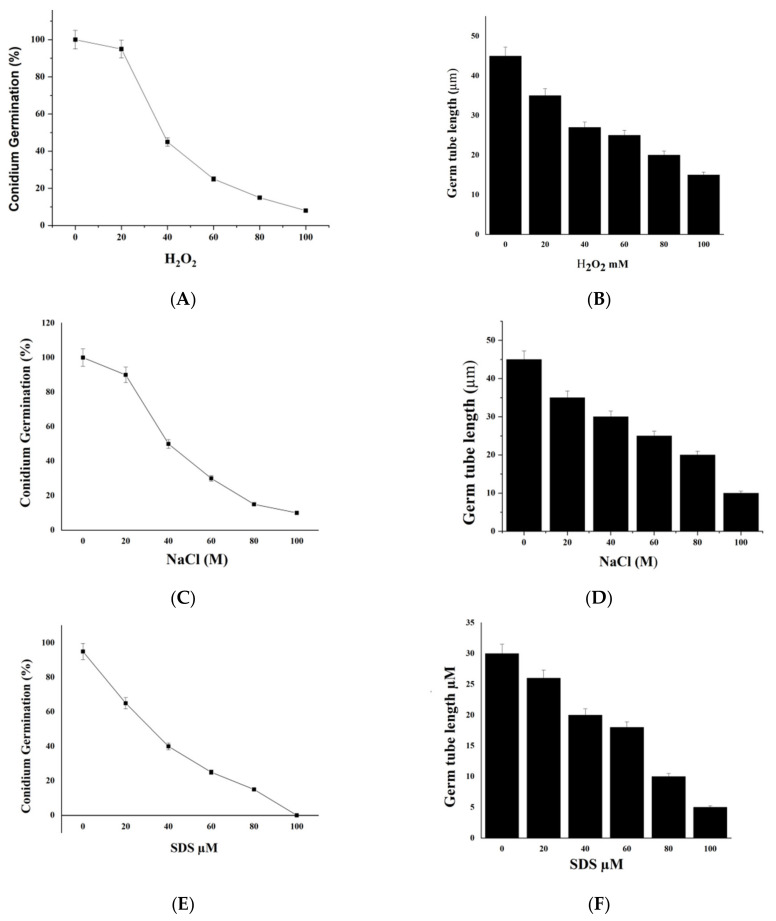

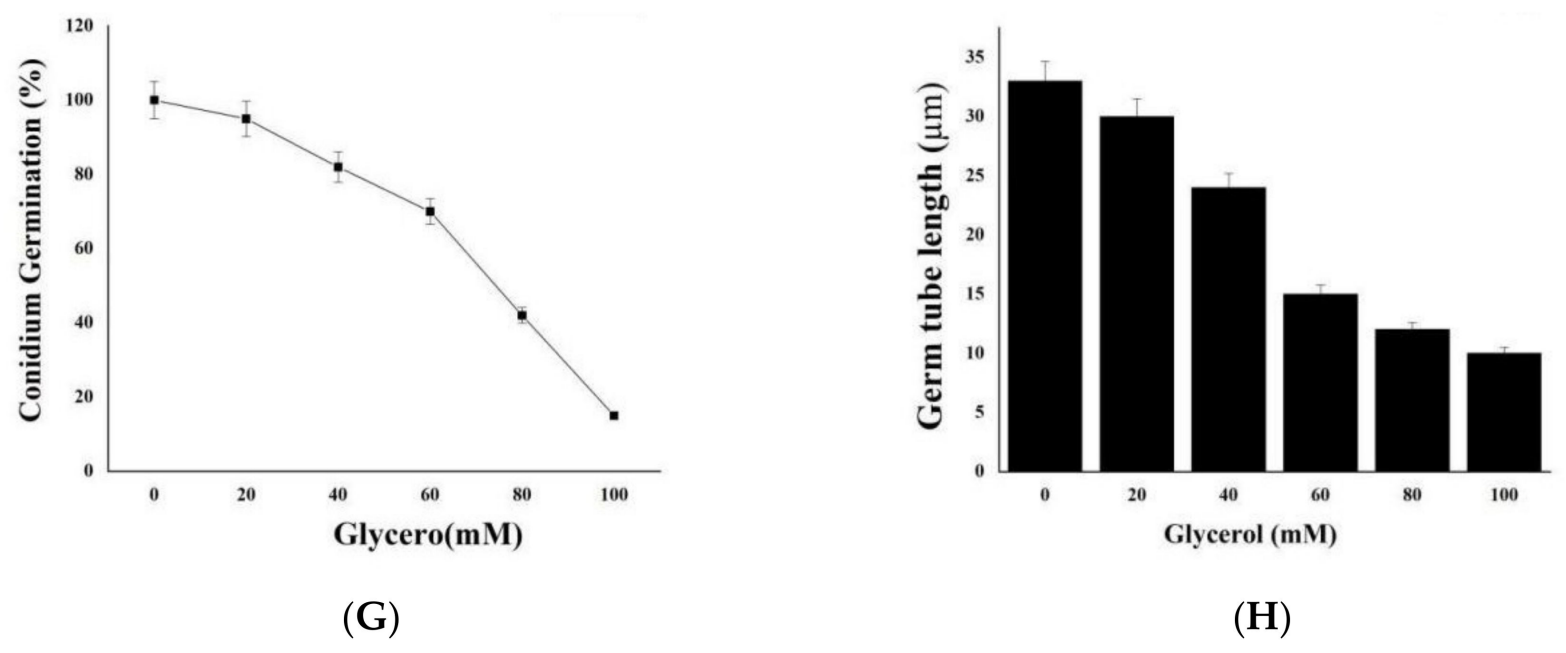
The effect of exogenous addition of environmental stresses on conidium germination and germ tube length of *Magnaporthe oryzae* at 6 h post-incubation. Additionally, the effect of various concentrations of NaCl (**A**,**C**,**E**,**G**) conidium germination and (**B**,**D**,**F**,**H**) germ tube length.

We added different concentrations of environmental stresses of H_2_O_2_, glycerol, SDS, and NaCl (0, 20, 40, 60, 80, and 100 mΜ) to the conidial suspension. Further, we dripped twenty microliters of the mixture from each treatment onto a microscope cover glass with wet filter paper and incubated at 28 °C in darkness. We examined the conidium germination and germ tube length at six hpi. The observable results indicated that all the chemical stresses tested reduced the conidium germination with a more substantial effect observed with increased concentration of the chemicals (Figure 7A,C,E,G). Additionally, the chemicals affected germ tube elongation, with the effects becoming more pronounced with the increase of the chemical concentration (Figure 7B,D,F,H).

### 2.8. DL76 Reduced M. oryzae Adaptation to the Environment and Contributed to Cellular Response and Abiotic Stresses

**Figure 8:**
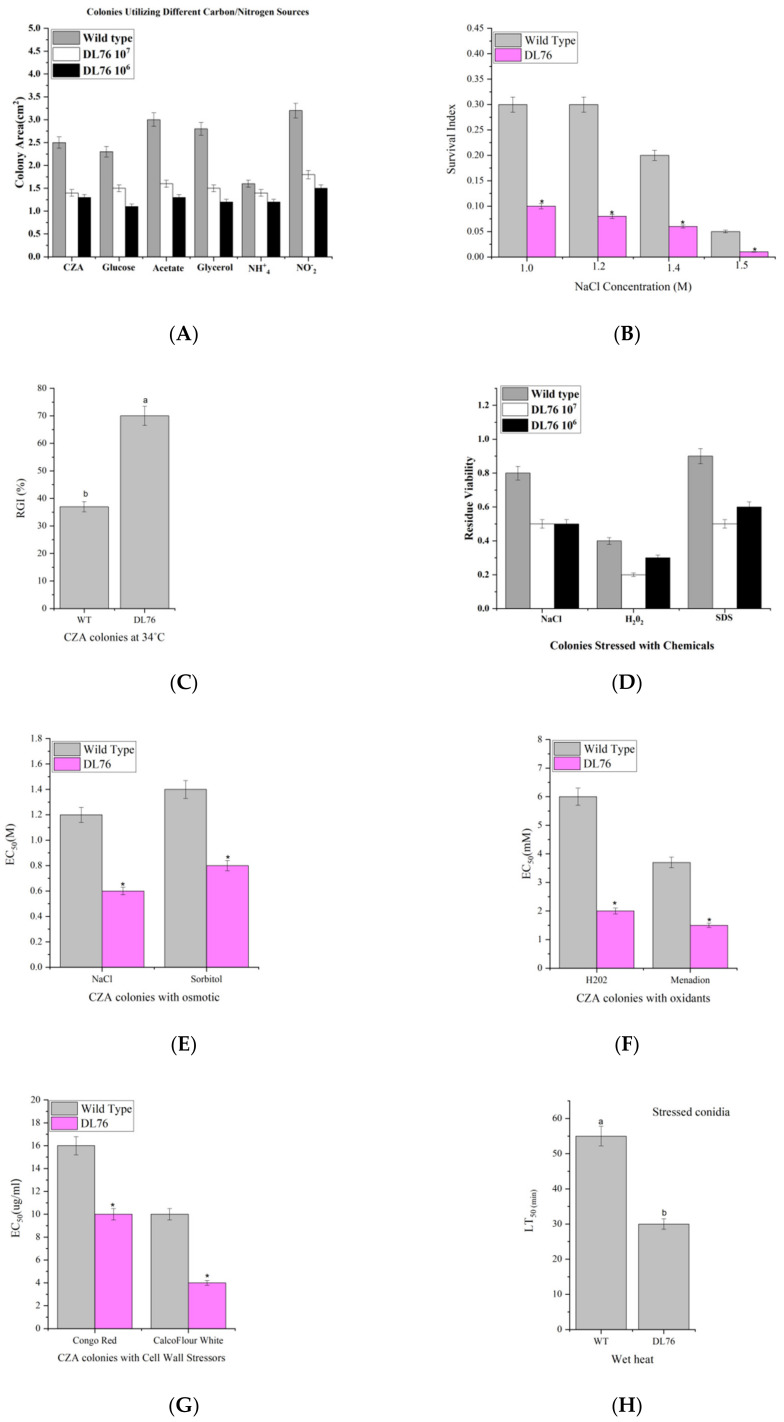

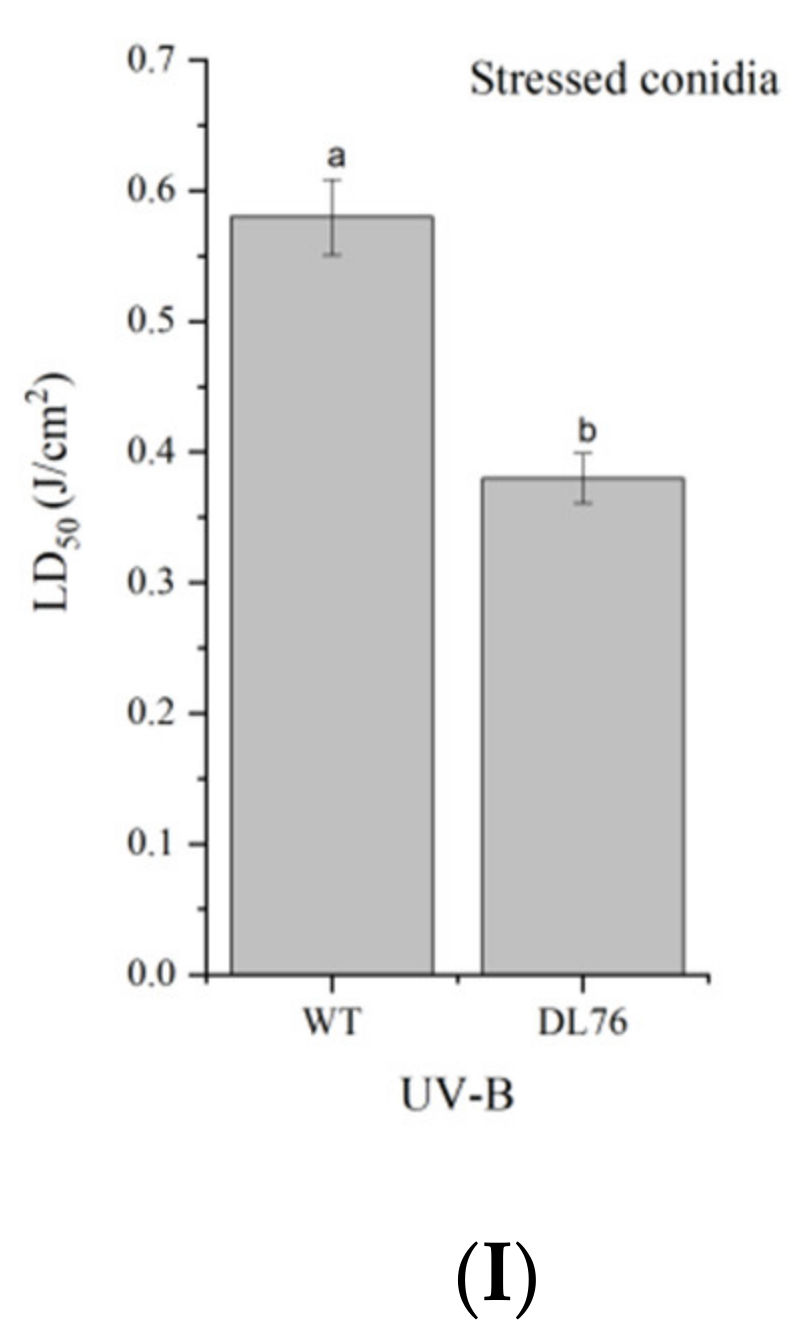
(**A**) represents colony sizes on minimal CZA (3% sucrose as mere carbon and 0.3% NaNO_3_ as mere nitrogen) and CZA-derived media [altered carbon: glucose, glycerol, acetate; altered nitrogen: NaNO_2_, NH_4_Cl and (**B**) The conidial survival indices of the tested strains under the hyperosmotic stresses (**C**) Relative growth inhibition (RGI) of a fungal colony at 34 °C. (**D**) represents residue viability of conidia after 24 h incubation at 25 °C on germination medium supplemented with NaCl (1.2 M), H_2_O_2_ (4 mM), or SDS (0.04%). (**E**–**G**) EC_50_s estimated for two oxidants, two osmotic agents, and two cell wall stressors to suppress 50% colony growth at 25 °C, respectively. (**H**,**I**) Median lethal time (LT_50_) for conidial tolerance to wet-heat stress at 45 °C and median lethal dose (LD_50_) for conidial UV-B resistance. Error bars represent the SEs of three replicates. Different letters indicate significant differences (*p* < 0.05) and * Significant difference (*p* < 0.05) based on one-way analysis of variance followed by the least significant difference test.

DL76 grew significantly slower than the control strains during the 8-day incubation at 25 °C on minimal CZA (Czapek agar) and 5 CZAR-derived media. The final size of the emerging colony was reduced by 46–60% on the sucrose carbon source, glucose, acetate, or glycerol and 32–46% on the nitrogen source of NO^−^_2_ or NH^+^_4_, and affected more by the starvation of carbon than nitrogen (Figure 8A). Consequently, we assayed the osmotic agents (NaCl) for their effects on the colony growth of each strain on the CZA Petri dish (Figure 8B) using the spotting method for colony initiation. We observed a significant difference between wild-type and DL76, based on the survival indices. Regarding the wild-type indices, germination was reduced from 0.3 to 0.05, whereas in DL76, it was 0.2 to 0.03. Hence, DL76 was more sensitive and less tolerant to stressful chemical NaCl than the wild-type. The stress tolerance of phytopathogens to osmotic stressors plays an essential role in pathogenicity [23,24]. Continuing using the spotting method, compared to wild type, DL76 was more sensitive to 34 °C with 27.5% during colony growth (Figure 8C). We used a sensitive concentration to assess the effects of NaCl, H_2_O_2_, and SDS on conidial germination. The results indicated that DL76 was significantly less than the wild-type, thus suggesting that DL76 is less tolerant to stressful chemicals (Figure 8D) based on the residual viability (germination). Further, we estimated EC_50_s for two oxidants, two osmotic agents, and two cell wall stressors to suppress 50% colony growth at 25 °C, respectively (Figure 8E–G). We initiated all colonies with the spotting method. In the two oxidants, H_2_O_2_ and menadion, DL76 decreased significantly and was more sensitive to all the chemicals (Figure 8E) than wild-type. In the two osmotic agents, NaCl and sorbitol, DL76 decreased significantly and was more sensitive to all the chemicals (Figure 8F) than wild-type. Comparing the colonies, DL76 was more sensitive compared to wild-type Guy11 (Figure 8G). In addition, conidial thermotolerance and UV-B resistance were reduced in DL76, as indicated by LT_50_ (min) and LD_50_ (J/cm^2^) (Figure 8H,I). The data indicates DL76 responses to oxidative, hyperosmotic, cell wall disturbing, thermal, and UV-B resistance.

### 2.9. DL76 Is Essential for Pathogenic Development in M. oryzae and Responsible for Appressorium-like Structure Formation

**Figure 9:**
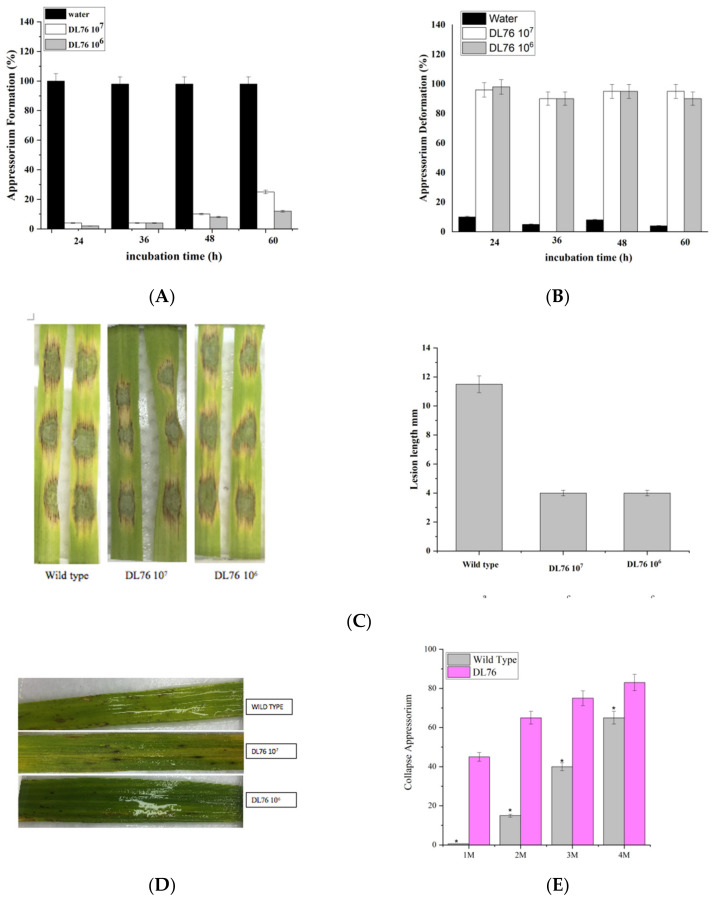
(**A**) is the statistical analysis of the appressorium like structure formation rate. More than 100 hyphal tips were observed each time. (**B**) is the appressorium deformity rate was measured and statistically analyzed. (**C**) Pathogenicity of conidia on isolated leaves (**D**) is the pathogenicity assay on abraded rice plant leaves. The tested strains were incubated on isolated rice leaves, and their virulence was evaluated 5 days after incubation. (**E**) is the measurement of collapsed appressoria. At least 100 appressoria were observed at each glycerol concentration. Error bars represent SD. * Significant difference (*p* < 0.05) based on one-way analysis of variance followed by the least significant difference test.

We observed that DL76 promotes fewer appressorium formation and suppresses appressorium formation in the previous results at both concentrations of 1 × 10^7^ CFU/mL and 1 × 10^6^ CFU/mL. We further checked if the two concentrations would produce appressorium-like structures by hyphal tips. We used four incubation times (24, 36, 48, and 60 hpi); at 24 hpi, DL76 in both concentrations generated fewer appressorium-like structures when compared with water. DL76 generated fewer appressorium-like structures than water as the incubation time increased from 24 hpi to 60 hpi (Figure 9A). Similarly, most of the appressorium-like structures formed by DL76 were abnormal and swollen compared to water. The deformation rate was higher in DL76 than in water (Figure 9B). Further, to investigate the pathogenic role of DL76 in *M. oryzae*, we inoculated 20 μL drops of conidial suspension from Guy11 and treated them with DL76 on wounded rice leaves.

The field and in vitro experiments indicated that DL76 significantly inhibits the growth of rice blast pathogen (Figure 9C). The wild-type Guy11 was fully pathogenic with extendible necrotic lesions, whereas DL76 caused small necrotic lesions on the inoculated site. The leaf lesion diameter in the DL76 group was 4.2 mm, whereas in wild type Guy 11 was 11.5 mm. We also compared the DL76 inhibitory pathogenicity against rice blasts with the control group. The lesion diameter on isolated leaves of the treatment group was smaller than that of the control group. This phenomenon indicated that the sterilized culture filtrate of DL76 had a strong ability to suppress the pathogenicity of *M. oryzae*.

On the other hand, we evaluated the DL76 inhibitory properties against rice blasts. We sprayed rice seedlings with five-fold dilutions of bacterial PDB cultures with 1 × 10^7^ and 1 × 10^6^ CFU/mL 36 h before inoculating with *M. oryzae*. The results indicated that the preventive application of DL76 at both concentrations effectively controlled rice blast disease. Further, we observed rare tiny lesions on rice leaves when we compared DL76 with the control group. The average lesion quantity on living leaves of the control group was 36 lesions per leaf compared, while the average lesion quantity of treatment was 15–20 lesions per leaf treated with DL76 (Figure 9D). This outcome informed that the sterilized culture filtrate of DL76 had a strong ability to suppress the pathogenicity of *M. oryzae*.

Similarly, we examined the appressorium turgor in the DL76 and wild-type Guy11 to further elucidate the mechanism underlying virulence in the DL76 in *M. oryzae*. Surprisingly, the appressoria of the DL76 observed a reduced collapse rate in 1, 2, 3, and 4 M glycerol compared to the wild-type. There were significant differences between the generated turgor pressures of the tested strains at these concentrations. For example, at 1 M glycerol, the appressorium collapse rate was 0.8% in wild-type Guy11 but was 45% in DL76. The collapse rates gradually increased from 0.6% to 65% in wild-type Guy11 and 45% to 83.5% in DL76, respectively, at 4 M glycerol (Figure 9E). The proportion of collapsed appressoria increased with the external glycerol concentration suggesting that DL76 reduces defects in maintaining appressorium turgor.

### 2.10. DL76 Is Involved in Down-Regulating the Expression of Oxidation and Osmotic Genes in M. oryzae

**Figure 10:**
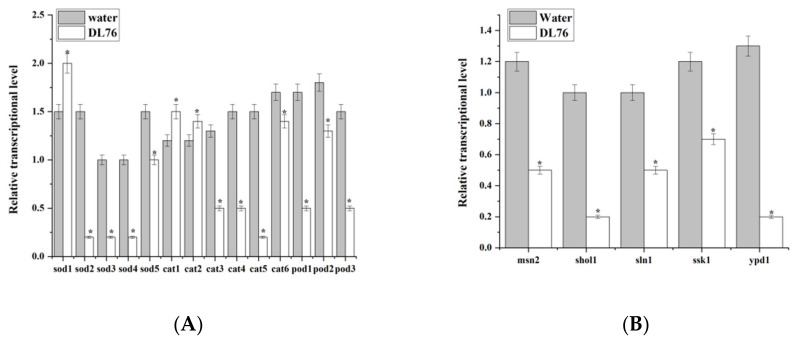
Transcription oxidative and osmotic involved genes in *Bacillus subtilis* DL76-treated conidia of *Magnaporthe oryzae*. Conidia were treated with sterile water or DL76 (1 × 10^7^ CFU/mL) for 2 h, and then, total RNA was extracted. Reverse transcription quantitative PCR was used to measure transcription. (**A**) Oxidative genes used to measure transcription level. (**B**) Osmotic genes used to measure transcription level. * Significant difference (*p* < 0.05) based on one-way analysis of variance followed by the least significant difference test.

In contrast, many of the examined genes were down-regulated by DL76 versus water under oxidative stress; RT-qPCR examined 14 genes. The expression level of 11 genes was significantly down-regulated by DL76 in *M. oryzae* than in water (Figure 10A). The transcript levels of *sod1*, *cat1*, and *cat2* were significantly increased in DL76. Under osmotic stress, the transcriptional level of five genes *msn2*, *sln1*, *shol1*, *ssk1*, and *ypd1* were examined. Compared to water, all five osmosensitive genes were down-regulated in *M. oryzae* by DL76 (Figure 10B). 

## 3. Discussion

Few potential biocontrol strains of rice blast suppression have been isolated from the plant rhizosphere in recent years [25,26]. Several species of bacteria, especially Bacillus, are known as biological control agents that suppress several phytopathogenic fungi due to cell wall-degrading enzymes and other anti-fungal metabolic creation [27,28,29]. Several studies observed that Bacillus strains could stimulate plant growth and disease resistance. Antecedent *B. subtilis* strains have been used as biological control agents, where they played a significant agricultural role in the biocontrol of several plant pathogens, including *M. oryzae* [30,31]. *B. subtilis* activity relies on unique biological control mechanisms that *Bacillus spp.* can compete for colonization sites in plants to form bio-films and enhance the provision of nutrients and phytohormones. It improves the interaction of pathogen and plant and suppresses the pathogen growth by antibiotics (Surfactin, Iturin, and Fengycin), toxins, and bio-surfactants. The mechanism associated with bacteriolysis involves the production of extracellular cell wall degrading enzymes, such as chitinase and β-1,3-glucanase [32,33]. Several Bacillus strains antagonistic to fungi have been isolated from soil; however, we isolated *Bacillus subtilis* DL76 from the field rice rhizosphere in this study [34]. Our results observed that culture filtrate of DL76 obtained a biocontrol deficiency of 75% under greenhouse conditions. Furthermore, the valuation of the antifungal activity of DL76 under different concentrations of the colony-forming unit was found to have a significant suppression growth of *M. oryzae*. The stability experiment suggested that the active metabolites have substantial stability for high temperature and acid, not alkaline conditions. This indicates that high stability is necessary for developing antifungal metabolites as a biocontrol agent. Similarly, *B. subtilis* DL76 achieved logarithmic growth after 8 h of cultivation at a given fermentation condition. The strongest antifungal activity was obtained during the stationary phase 40 h after inoculation. We also observed a direct association between inoculation time and antifungal activity. That is, an increase in the inoculation time increases the anti-fungal activity. In the previous study, the antibiotics usually start to be produced at the end of the exponential growth phase. It reaches maximum concentration after cell growth has ceased, which conforms with the production kinetics of the strain DL76. Germination of conidia and the formation of appressorium are two crucial processes of *M. oryzae* during plant infection. In conidium development, the concentration of 1 × 10^7^ CFU/mL of DL76 suppressed conidia germination at eight hpi. In contrast, a lower concentration (1 × 10^6^ CFU/mL) allowed some conidia to germinate, and the concentration of 10^5^ CFU/mL observed no suppression effect at 24 hpi. The concentration of 1 × 10^7^ CFU/mL of DL76 completely suppressed conidium germination at 24, 36, 48, and 60 hpi with an extended time. Furthermore, the germ tubes of conidia treated with DL76 at high concentration were remarkably shorter than those treated at low concentration or with water. We monitored appressorium formation and the turgor pressure inside the appressoria. At both concentrations of 1 × 10^7^ and 1 × 10^6^ CFU/mL appressorium formation was suppressed, and no appressorium was observed. However, lower concentration permitted appressorium formation. These results suggested that *B. subtilis* DL76 at lower concentration does not affect the eventual appressorium formation and turgor pressure inside the appressoria. It indicates that the germination of conidia was delayed and suppressed and the fermentation filtrate of DL76 hindered the formation of appressorium. Therefore, we can conclude that the filtrate of DL76 can suppress the formation of infectious structures of *M. oryzae* in vitro.

We noted that the conidia treated with the culture filtrate of DL76 lost pathogenicity in the pathogenicity test. In contrast, the lesion of the wild type was bigger than that of DL76. The number of lesions in DL76 was much lower than that in the wild type. Conclusively, our results inform that in the infection situation, the pathogenicity of *M. oryzae* was significantly suppressed due to DL76-treatment. Furthermore, we suggest that DL76 can suppress growth, germination, and appressorium formation in vitro. That is the pathogenicity of *M. oryzae* in vitro.

Besides, various well-conserved signaling pathways have been verified to be involved in *M. oryzae* germ tube-derived appressorium formation and penetration [35,36]. The regulation mechanisms of appressorium formation and appressorium-like structure development differ in structure-function during penetration and pathogenicity [37,38,39]. In this research, we found that DL76 does not affect the formation of appressoria at 1 × 10^7^ and 1 × 10^6^ CFU/mL. We examined for appressorium-like structure (ALS) to understand its reasons. The results indicated that DL76 completely suppressed the appressorium-like structure at hyphal tips on the hydrophobic surface, and most of the appressorium-like structures formed by DL76 were deformed. Additionally, DL76 dramatically lost its ability to infect rice leaves and cause disease symptoms. This led us to examine the appressorium turgor pressure. We found out that the collapse rate of appressoria of DL76 was remarkably higher than that of wild-type Guy11 at all concentrations, implying that appressorium turgor in *B. subtilis* DL76 was remarkably decreased.

Cell wall integrity (CWI) plays an essential role in responding to external stress, fungal growth, and pathogenicity. The fungal cell wall provides mechanical protection against attack from the host during host-pathogen interactions. Thus, maintaining cell wall integrity is critical for phytopathogens to establish disease on their host [40,41,42]. This study observed that the *B. subtilis* DL76 was a more sensitive cell wall perturbing agent and showed gradient reductions in tolerance to high temperature, oxidation, high osmolarity, and cell wall perturbation during colony growth. Moreover, the relative transcript level of stress-responsive genes was examined. We found that in oxidation conditions, the expression level of the stress-responsive genes was significantly reduced by DL76 except for *Sod1*, which showed an increased expression level in DL76. However, in osmotic conditions, all the stress-responsive genes were remarkably reduced by DL76. 

## 4. Materials and Methods

### 4.1. The Evaluation of Antifungal Activity

In this experiment, we tested the inhibition of *M. oryzae* against the strain DL76. The strain DL76 was inoculated to LB liquid medium and incubated at 28 °C on a rotary shaker Thermo Fisher Scientific, Waltham, MA, USA). For 48 h, followed by lysine and removal of bacterial cells. The supernatant was filtered through a 0.22 μm membrane filter to remove the bacteria cells, and the filtrate was mixed with solid LB at different concentrations (1 × 10^2^, 1 × 10^3^, 1 × 10^4^, 1 × 10^5^, 1 × 10^6^, 1 × 10^7^ colony-forming units (CFU) per mL). Lastly, the mixture was poured into Petri dishes. The fungus mycelia plug of Guy11 was transferred to the center of the medium and incubated at 28 °C in the incubator (Fisher Scientific, Waltham, MA, USA).

### 4.2. The Stability of PH and Temperature of Filtrate on the Growth Suppression of M. oryzae

We prepared the sterilized culture filtrate of DL76 to determine the stability of pH and temperature against *M. oryzae*. We took out 70 mL to be placed in the seven Vitro, adjusted to various pH values to 1, 3, 5, 7, 11, 13, respectively, and stored in the refrigerator at 4 °C. After 24 h, the samples were readjusted to pH 7 and took out 1.5mL from each of the Vitro to mix up with molten potato dextrose agar (PDA) mediums making filtrate of concentration to 6.25 μL/mL (1:160, *v*/*v*). The fungus cake of Guy11 was transferred to a Petri dish. After incubation at 28 °C for seven days, the growth of Guy11 was observed, and the colony diameter of Guy11 was measured to assess the growth inhibition rate compared to control. Regarding the temperature test, the sterilized and broth culture filtrate were exposed to 4 °C, 28 °C, 37 °C, 60 °C, 80 °C, 100 °C, and 121 °C for 30 min. Similarly, this test set was repeated three times after cooling the sample to room temperature. The relative suppression rate was described as follows;

Relative inhibition (%) = [(K_1_ − K_2_)/K_1_] × 100

where K_1_ = *M. oryzae* colony diameter in the control and R_2_ = *M. oryzae* colony diameter in the dual culture Petri dishes with a mixture of sterilized culture filtrate of DL76. 

### 4.3. Correlation between Cell Growth and Anti-Fungal Activity

We inoculated *Bacillus* *subtilis* DL76 into 500 mL of potato dextrose liquid medium and incubated it at 28 °C, 180 rpm for 108 h. During the incubation, samples were taken at eight hours to measure OD_600_ (Eppendorf BioPhotometer, Marshall Scientific LLC, Hampton, NH, USA) and antifungal activity against *M. oryzae*. Further, we used 1.5mL from each vitro to mix with molten potato dextrose agar mediums making a filtrate of 6.25 μL/mL (1:160, *v*/*v*) concentration. The fungus cake of Guy11 was transferred to the Petri dish (and incubated at 28 °C for seven days). The growth of Guy11 was observed, and its colony diameter was measured to assess the growth suppression rate compared to the control.

Relative inhibition% = [(K_1_ − K_2_)/K_1_] × 100

K_1_ represents *M. oryzae* colony diameter in control, and K_2_ represents *M. oryzae* colony diameter in the dual culture plates with sterilized culture filtrate of DL76 mixed with PDA.

### 4.4. Effect of the Supernatant at Different Concentrations on Conidium Germination, Appressorium Formation, Germ Tube Length, Turgor Pressure, Infection Process, Vegetative Growth and Conidiation, and Cell Activity

#### 4.4.1. The Conidia Germination Test

We used 10-milliliters of water to scrape the conidia from a 10-day-old *M. oryzae* conidia-producing medium adjusted to a concentration of 1 × 10^5^ conidia/mL after filtration with two layers of lens paper. Further, we added one milliliter of sterile water, LB broth, and HS24 as the control groups and 1 mL of DL76 at different concentrations (1 × 10^2^, 1 × 10^3^, 1 × 10^4^, 1 × 10^5^, 1 × 10^6^, 1 × 10^7^ CFU/mL) to each tube. Fifty micro-liters of conidia suspension were dropped on the hydrophobic cover slide. We observed the appressorium formation under a ZEISS fluorescence microscope (Tokyo, Japan) after incubation at room temperature in a moist Petri dish for 8, 12, 24, 36, 48, and 60 h. We then selected 100 conidia to compute the germination rate. The experiment was repeated three times.

The formula for the germination rate was as follows:

Germination rate (%) = [K_1_/K_2_] × 100%

where K_1_ = the number of germinated conidia and K_2_ = total conidia.

#### 4.4.2. The Appressorium Formation

We scraped conidia with sterilized water and culture filtrate from a 10-day-old *M. oryzae* conidia-producing medium, then diluted the suspension to 1 × 10^5^ conidia/mL. Afterward, fifty micro-liters of conidia per droplet were dripped on a hydrophobic cover slide. We observed the appressorium formation under a ZEISS fluorescence microscope (Tokyo, Japan) after incubation at room temperature in a moist Petri dish for 8, 12, 24, 36, 48, and 60 h. The experiment was repeated three times.

The formula for appressorium formation rate was as follows:

Formation rate of appressorium (%) = [V_1_/V_2_] × 100%

V_1_: the number of conidia that formed appressorium;

V_2_: the number of total conidia.

### 4.5. Environmental Stresses at Different Concentrations of SDS, H_2_O_2_, Glycerol, and NaCl (0, 20, 40, 60, 80, and 100 µΜ) Were Added to the Conidial Suspension

Twenty microliters of the mixture from each treatment were dripped onto a microscope cover glass with wet filter paper and incubated at 28 °C in darkness. Conidium germination and germ tube length were examined at six hpi.

### 4.6. Measurement of Growth and Conidiation Parameters

Aliquots (1ml) of 1 × 10^6^ conidia/mL suspension treated with DL76 at various concentrations were spotted centrally on the Petri dishes (9 cm diameter) of nutrition-rich SDAY (4% glucose, 1% peptone, and 1.5% agar plus 1% yeast extract), minimal CZA and eight CZA-derived media. The derived media were prepared by excluding 3% sucrose (carbon starvation) from standard CZA, replacing 3% sucrose with 3% of glucose, galactose, glycerol, or acetate (NaAc) as the sole carbon source, and replacing 0.3% NaNO_3_ with 0.3% of NaNO_2_ or NH_4_Cl as sole nitrogen source, respectively. During the eight-day incubation at 25 °C and 12:12 h, colony diameters were cross-measured daily to compute the colony area as a growth rate index.

Further, 100 mL aliquots of 1 × 10^7^ conidia/mL suspension treated with DL76 at different concentrations were evenly spread on SDAY Petri dishes and incubated for 7-days at 25 °C and 12:12 h to assess the sporulation capacity of each strain. From the 3rd day onwards, 5-mm diameter colony discs were cut off daily from the Petri dishes, and the conidia on each disc were washed into 1 mL of 0.02% Tween 80 by vortexing. We removed the hyphal debris by filtration, determined the conidial concentration with microscopic counts, and converted it to the number of conidia per 2 cm colony. Further, the fungal mass samples taken from the colonies were evaluated under the microscope (Olympus, Tokyo, Japan) to observe possible morphological changes in their conidiophores during the incubation.

### 4.7. Assaying Cellular Responses to Chemical and Environmental Stresses

SDAY Petri dishes overlaid with cellophane were spread with 100 mL aliquots of conidial suspension treated with DL76 at different concentrations to produce uniform cultures after 3-day incubation at 25 °C. We cut a 5 mm diameter fungal mycelial discs from the culture of each strain and placed them centrally onto the Petri dishes (90 mm diameter) of 1/4 SDAY supplemented with the gradients of NaCl (0.4–2 M), menadione (2–8 mM), H_2_O_2_ (20–80 mM), Congo red (0.5–3 mg/mL), SDS (0.02–0.12%) and carbendazim (0.4–2 mg/mL), respectively. After 6-day stressful incubation at 25 °C and 12:12 h, the diameters of all colonies in the control and stress treatments were cross-measured to compute their net area increases.

Cellular responses to stressful chemicals were assayed by spotting aliquots of 1 μL of a 10^7^ conidia/mL suspension onto the Petri dishes of CZA alone (control) or supplemented with the respective gradients of menadione (0–40 μM), H_2_O_2_ (04 mM), NaCl (0–0.7 M), sorbitol (0–1.5 M), Congo red (0–50 μg/mL), and calcofluor white (0–20 μg/mL), followed by seven days of incubation at 25 °C. Chemical-free Petri dishes incubated at 34 °C for seven days were included as a heat stress treatment. All colony diameters were measured on day 7. An effective concentration (EC_50_) for each chemical to suppress 50% colony growth was estimated by modeling growth trends over the gradient. The heat stress estimated relative growth inhibition of fungal colonies (T_C_ − T_S_)/T_S_ × 100, where T_C_ and T_S_ denote colony diameters at 25 °C (control) and 34 °C, respectively.

Additionally, we assayed the conidial tolerances to oxidation, hyperosmolarity, and cell wall disturbance by spreading 100 mL aliquots of conidial suspension treated with DL76 at different concentrations onto the Petri dishes of germination medium (GM: 2% sucrose and 0.5% peptone plus 1.5% agar) supplemented with menadione (0.2 mM), H_2_O_2_ (4 mM), NaCl (1.2 M), Congo red (1 mg/mL) and SDS (0.04%), respectively. We assessed the conidial germination on each of the plates, stressed or not, using three counts of germinated and ungerminated conidia under a microscope after 24 h of incubation at 25 °C. We calculated the residue viability as the ratio of percent germination under each stress over that in control. Similarly, we used the previous protocol to assay the conidial tolerances to wet-heat stress of 15–120 Min at 45 °C and UV-B irradiation (weighted 312 nm) of 0.1–0.8 J/cm 2 in the Bio-Sun 11 chamber (Vilber Lourmat, Marnela-Valle’e, France). Additionally, after exposure to a specific intensity of heat or UV-B stress, conidia were incubated for 24 h at 25 °C under saturated humidity. Percent germination was determined using microscopic counts.

### 4.8. Assays for Stress Tolerance of Antisense Transformants

Hyperosmotic stress assay for each strain, 10-μL aliquots of spore suspension (1 × 10^6^ conidia mL^−1^) were spread evenly on the Petri dishes of Czapek’s agar supplemented with 0–1.5 M NaCl for different degrees of osmotic stress (Zhang et al., 2009). After 24-h of incubation at 25 °C, percent germination on the Petri dishes was determined above. We estimated the osmotic survival index as the ratio of the percent germination on the salt-inclusive Petri dish over that on the salt-free Petri dishes.

### 4.9. Experiment on Isolated Leaves

Rice seedlings were grown in an artificial climate chamber (30 °C) for 30 days. Rice leaves without apparent disease symptoms were placed in Petri dishes with a solution of 6-benzylaminopurine. Before that, every leaf was poked slightly with a needle making it more susceptible to *M. oryzae* infection. Regarding the treatment group: a five μL volume of *M. oryzae* conidia suspension (1 × 10^5^ conidia/mL) droplets was diluted with a sterilized culture filtrate of *Bacillus subtilis* DL76 and applied to the slightly punctured sites of the leaves. The inoculated leaves were incubated at 25 °C in the dark for 24 h.

### 4.10. RNA Extraction and Transcripts of Stress-Responsive Gene Transcription Analysis

The strain guy11 conidia co-cultured with sterile water or DL76 were collected at two hpi for RNA extraction. The Prime Script RT reagent kit (TaKaRa Bio Inc., San Jose, CA, USA) performed a reverse transcription test. Transcription of the stress-responsive gene was examined by reverse transcription-quantitative PCR (RT-qPCR) on an ABI 7500 Fast Real-Time System (Applied Biosystems, (AB Ltd., Lincoln, NE, USA) following previously established procedures (Guo et al., 2010). The primers used in this study were designed using the NCBI (https://www.ncbi.nlm.nih.gov accessed on 23 June 2021), as shown in Table 1.

### 4.11. Statistics and Data Analysis

All treatments were repeated at least three times, and >100 conidia were observed in each replicate. Data were analyzed with SPSS 17.0 (IBM Corporation) by one-way analysis of variance followed by means separation using the least significance difference (LSD) test (*p* < 0.05). The ratio of colony size or conidial germination rate under stress over control was defined as relative viability (V_R_). For each of the tested strains, the VR trends over the concentrations (C) of each chemical, the time lengths (T) of wet-heat stress, and the doses (D) of UV-B irradiation were fitted to the equation V_R_ = 1/[1 + exp (a + bx)]. Where x is C, D, or T, a and b are parameters to be estimated. When V_R_ = 0.5, the fitted equations gave solutions (−a/b) to effective concentration (EC_50_) of each stressful chemical required to suppress 50% colony growth and median lethal responses of conidia to heat (LT_50_, min) and UV-B (LD_50_, J/cm^2)^ stresses.

## 5. Conclusions

In summary, we have observed that *B. subtilis* DL76 can inhibit the growth, germination, and formation of appressorium in vitro and the pathogenicity of *M. oryzae* in vivo. DL76 significantly suppressed the conidial germination and appressorial formation of *M. oryzae*. Moreover, DL76 might effectively inhibit infection by influencing the formation of the infectious structure of *M. oryzae.* Conidial thermotolerance and UV-B resistance were reduced in DL76 disruption. Furthermore, DL76 also reduced large parts of conidial tolerances to both oxidative and cell wall perturbing stresses. Finally, the transcriptional level of DL76 was down-regulated both under osmotic and oxidative stress. We can conclude that *Bacillus subtilis* DL76 significantly contributes as a candidate biological control agent against *M. oryzae* and has furthered the progress of pathogenesis research.

## Figures and Tables

**Table 1:** Primers used for quantitative real-time PCR.

Involved in Response to Oxidative Stress		
sod1	GCGGCTTCCACATCCACACCTTTG	GGTCCAGCGTTGCCAGTCTTGAG
sod2	CCAGTGTTTGGCATTGACATG	TCAGCCGTCTTCCAGTTGATG
sod3	TCTCCGGCAAGATTATGGAGC	TTGGCGTCATTCTTGGCCT
cat1	CCTCTGACGTTGGCGGCCCTTTC	CCGTGTCCGTGCTGCCTCGTG
cat2	CCGTCTGGGCATCAACTGGGAAG	GCTGGGCGTGGTCGTGGTAG
cat3	TCAAGTCGGTTCAGGAGATGGAG	TTGTTGCGTCTTCAATCGGAGTG
cat4	GAGGAGCCCAGCAACGCACAAGAG	CTGAGGACGACAAGGCCGCCATTC
cat5	GCTGGGCTGATCTGCTGGTCCTTG	TCCTTGCTGTAACGGTGGCTGTCG
cat6	CGGCTGCGGTGTCTTGTCCATAC	CCTTGTCGGCGTTCTGGCGAAG
**Involved in Response to Osmotic Stress**		
sho1	AGCGTCAACAACCTCGTCTAC	AGGCGTGGAGTTCTCAAAG
sln1	TCGGTGCCATCACTCCTTCCAACG	GCGGACGAGCAATGCCAACAGC
ssk1	AGAACTTTAGCACCGAGCCCTTTC	AGAAGCAGCAGCAGACGATTGG
ypd1	TGACCAGGTCGAGGAGACGTTTGC	TCGGCGTCGGGTTCTGATGAGC
msn2	GCCCGCCACGCCCATCTAC	ACCGAGGTCTCAACCGAGTCAAAC

## Data Availability

Not applicable.
